# Patient-related diagnostic delay and risk of unfavorable treatment outcomes among pulmonary tuberculosis patients at the antituberculosis center of Brazzaville, Republic of Congo

**DOI:** 10.1038/s41598-026-50300-z

**Published:** 2026-05-22

**Authors:** Breli Bonheur Ngouama, Mita Naomie Merveille Dello, Freisnel Hermeland Mouzinga, Darrel Ornelle Elion Assiana, Jean Claude Djontu, Franck Hardain Okemba Okombi, Franck Hardain Okemba Okombi, Michel Illoye Ayet, Jeannhey Christevy Vouvoungui, Lemercier Khunell Siele, Viny Anzi Elenga, Alain Maxime Mouanga, Alain Brice Vouidibio Mbozo, Francine Ntoumi

**Affiliations:** 1 Fondation Congolaise pour la Recherche Médicale, Brazzaville, Republic of Congo; 2 Faculté des Sciences et Techniques, Université Marien Ngouabi, Brazzaville, Republic of Congo; 3 Centre Antituberculeux de Brazzaville, Brazzaville, Republic of Congo; 4 Programme National de Lutte contre la Tuberculose, Brazzaville, Republic of Congo; 5 Faculté des Sciences de la Santé, Université Marien Ngouabi, Brazzaville, Republic of Congo; 6 Institute of Tropical Medicine, University of Tübingen, Tübingen, Germany

**Keywords:** *Mycobacterium Tuberculosis*, Brazzaville, Diagnosis delay, Treatment outcome, Diseases, Health care, Medical research, Risk factors

## Abstract

Tuberculosis (TB) remains a major public health concern worldwide. Early diagnosis is crucial to reduce TB transmission and improve treatment outcomes. This study assessed patient-related diagnostic delay and their impact on the treatment outcomes among pulmonary tuberculosis patients (PTB) at the Antituberculosis center in Brazzaville. We conducted a prospective cohort study from July 2023 to August 2024. Sociodemographic, clinical characteristics, patient-related diagnostic delays delay (short ≤ 30 days, prolonged > 30 days), and treatment outcomes were recorded. Logistic regression models were used to identify risk factors, reporting crude and adjusted odds ratios (OR, AOR) with 95% confidence intervals (CI). A p-value < 0.05 was considered significant. A total of 313 patients was included (median age: 34 years, range 24–41); 295 (94.2%) were newly diagnosed, and 16 (5.1%) were HIV-positive. Men accounted for 69% of cases, and the age group 24–44 was the most represented (55.9%). The median patient delay was 30 days (IQR 21–62), and 135 (43.1%) experienced prolonged delays. Multivariate analysis showed that residence in Mfilou district was associated with longer delays (OR = 2.77, 95%CI: 1.22–6.30; *p* = 0.004), whereas diabetes mellitus was linked to shorter delays (OR = 0.15, 95%CI: 0.02–1.13; *p* = 0.008). Although patient-related diagnostic delay was not significantly associated with treatment outcomes, patients with delays > 30 days had higher odds of death (OR = 2.30, 95%CI: 0.7–7.3) and treatment failure (OR = 5.4, 95%CI: 0.8–66.4). A high median patient-related diagnostic delay of 30 days was observed in Brazzaville. Residence in peripheral districts and diabetes mellitus status were significant predictors of delay. Although not statistically significant, prolonged patient delays tended to be associated with higher risks of death and treatment failure. Strengthening early case detection and promoting prompt healthcare-seeking and diagnosis among symptomatic individuals are critical for reducing TB diagnostic delays and improving treatment outcomes.

## Introduction

 Tuberculosis (TB) is an infectious disease caused mainly by *Mycobacterium tuberculosis* and remains the leading cause of death from a single infectious agent worldwide. In 2024, TB was responsible for an estimated 1.23 million deaths, including 1.08 million among HIV-negative individuals and 150,000 among people living with HIV. Although this represents a decline compared with 2023 (1.25 million), 2022 (1.32 million), 2021 (1.42 million), and 2020 (1.40 million), and even lower than the pre-COVID-19 level of 2019 (1.34 million), TB continues to pose a significant global health challenge^1^. While the global burden of TB is declining slowly, progress in controlling the disease and mitigating its consequences could be accelerated through the implementation of effective strategies that emphasize early diagnosis coupled with prompt initiation of treatment. Early diagnosis and treatment together form a key strategy for controlling TB^2,3^. Delays in diagnosis increase the risk of transmission, disease progression, and mortality, while also generating psychological distress and financial burdens for patients and their families. Previous studies have highlighted that late detection of TB not only worsens individual outcomes but also threatens community health and health system performance^4–6^. Despite advances in knowledge and diagnosis tools, delayed case detection remains common, particularly in Sub Saharan Africa, where health systems are often fragile^7–11^.

The Republic of Congo is classified among the 30 countries with the highest TB burden, with an estimated incidence of 302 cases per 100,000 inhabitants in 2024^1^. However, TB case detection largely relies on passive case finding, where patients are diagnosed only after presenting to health facilities with symptoms. This approach increases the likelihood of prolonged patient delays before diagnosis, thereby contributing to continued transmission and poor outcomes. To date, no study in the Republic of Congo has specifically assessed patient-related diagnostic delay in TB and their association with treatment outcomes. Understanding this delay and its determinants is essential to inform interventions that improve early case detection and strengthen the national TB program. Therefore, this study aimed to (i) estimate the median time to TB diagnosis, (ii) identify sociodemographic and clinical risk factors associated with prolonged patient-related diagnostic delay, and (iii) examine the impact of patient-related diagnostic delay on treatment outcomes among patients with pulmonary TB at the Antituberculosis Center of Brazzaville.

## Methods

### Study setting

The Republic of Congo is a high TB burden country with a population of approximately 6 million people. The study was conducted at the Antituberculosis Center of Brazzaville, the political and administrative capital, which hosts 35% of the population (2, 145, 783 inhabitants). In 2023, a total of 5, 757 TB cases (including pulmonary and extrapulmonary forms) were reported across approximately 25 TB centers in Brazzaville. All public health centers in the country provide free TB diagnostic and treatment services, including Directly Observed Therapy (DOTs), through a network of hospitals and health centers. This study was conducted at the Antituberculosis Center of Brazzaville, which reports approximately 40% of all TB cases in the city and is the sole center providing treatment for multidrug-resistant (MDR) TB patients. All 313 study participants were enrolled at this center.

### Study design and participants

A prospective cohort study was conducted between July 2023 and August 2024 at the Antituberculosis Center of Brazzaville to estimate patient-related diagnostic delay and its association with tuberculosis treatment outcomes. During the study period, 1,330 patients aged 8 to 80 years who consecutively presented. to the center with presumptive pulmonary tuberculosis symptoms were screened.

Eligibility was restricted to patients with pulmonary tuberculosis bacteriologically confirmed by Xpert MTB/RIF Ultra and with a complete questionnaire. Among the screened patients, 313 met all eligibility criteria. All 313 eligible patients consented to participate, were enrolled, completed the questionnaire, and were included in the final analysis. No eligible patient refused participation, and no questionnaires were incomplete or excluded; therefore, there was no loss between eligibility, enrollment, and analysis, and the numbers at each stage were identical (*n* = 313). After inclusion, patients were followed according to standard tuberculosis care. Any subsequent events (cure, treatment failure, death, or loss to follow-up) were recorded as treatment outcomes and analyzed as study results, rather than considered losses during enrollment.

Patient enrollment and data collection were carried out by clinicians working at the Antituberculosis Center of Brazzaville. Prior to study initiation, all personnel involved in recruitment and data collection received standardized training to ensure uniform application of eligibility criteria and consistency in data collection procedures.

### Data collection and definitions

Data were collected using a structured questionnaire covering sociodemographic and clinical characteristics. Participants were asked to report whether they had experienced key symptoms (cough, chest pain, fever, weight loss, hemoptysis, and night sweats) and to recall the duration of these symptoms.

For participants under 18 years of age, information on medical history and symptoms was obtained through interviews conducted with the patient and their parent or legal guardian, following parental consent and, where appropriate, child assent.

All questionnaires were administered by trained healthcare workers. Completed questionnaires were reviewed daily for completeness and internal consistency by the study supervisor before data entry. Collected data were securely stored and accessible only to authorized study personnel.

- **Patient-related diagnostic delay** was defined as the time interval (in days) between the self-reported onset of tuberculosis-related symptoms and the patient’s first presentation at the Antituberculosis Center of Brazzaville, where diagnostic evaluation, (clinical assessment and sputum sample collection for bacteriological testing), leads to confirmed diagnosis within a very short interval, generally on the same day or within 24 rarely 48 h. Given the organization of TB services (free of charge) in this setting, this interval represents the main component of the overall diagnostic delay. We considered 30 days as a cutoff for patient-related diagnostic delay, corresponding to the median delay observed in our study population as previously considered by^12^. Tuberculosis treatment outcomes and their related risk factors were described in our previous study^13^.

Treatment outcomes were monitored according to WHO definitions.

- **Treatment success** was defined as the sum of cured and treatment completed.
- **Unsuccessful outcome** was defined as the sum of treatment failure, death, and lost to follow-up.

Sputum samples collected at follow-up were examined using Ziehl-Neelsen staining for acid-fast bacilli, in accordance with the national tuberculosis program guidelines. Patients were followed throughout the treatment period as previously described^13^.

### Ethical considerations

Approval was obtained from the institutional ethics committee of the Fondation Congolaise pour la Recherche Médicale (Reference 045/CEI/FCRM/2023). All participants received full information about the study objectives and procedures and signed an informed consent form prior to inclusion, in accordance with principles of the Declaration of Helsinki. For children aged 8–17 years, assent was obtained along with written consent from parents or guardians. All data were anonymized to ensure participant confidentiality.

### Data management and statistical analysis

All analyses were performed using R (version 4.5.2; R Core Team, Vienna, Austria) within the RStudio integrated development environment (Posit Software, Boston, MA, USA). The main packages used included stats, MASS, and ggplot2 for visualization. Patient-related diagnostic delay was calculated as the number of days between symptom onset and TB diagnosis and categorized as ≤ 30 days (short delay) or > 30 days (prolonged delay). Because all participants had confirmed diagnosis at the time of inclusion, time-to-event analysis was not performed, and the outcome was treated as a binary variable. Associations between patient-related diagnostic delay and independent variables (age, sex, education, occupation, marital status, residence, HIV status, diabetes mellitus, TB history and treatment outcomes) were assessed.

Bivariate analyses were performed to estimate crude odds ratios (OR) with 95% confidence intervals (CI). Variables with *p* < 0.10 were included in multivariate logistic regression to calculate adjusted odds ratios (AOR). A p-value < 0.05 was considered statistically significant.

## Results

### Sociodemographic and clinical characteristics of the study population

Of the 1330 screened patients, 313 with pulmonary TB and complete questionnaires were included in the final analysis. Men accounted for 69.3% (217/313) of participants, with the age group 24–44 years being the most represented (55.9%). The median age was 34 years (IQR 24–41). The majority of patients (98.1%) had at least primary education, while six (1.9%) were illiterate. The most common districts of residence were Mfilou (16.6%), Talangai (16.0%), Makélékélé (15.3%), and Madibou (11.5%). Most patients were employed (85.6%) and single (93.9%). Sixteen patients (5.1%) were HIV-positive **(**Table 1**)**.

**Table 1:** Sociodemographic and clinical characteristics of the study patients.

Variables	Frequency *N* = 313	Percentage (%)
Sexe		
Male	217	69.3
Female	96	30.7
Age group		
< 24	71	22.7
24–44	175	55.9
≥ 44	67	21.4
Place of living		
Makelekele	48	15.3
Bacongo	28	8.9
Poto-Poto	9	2.9
Moungali	29	9.3
Ouenze	26	8.3
Talangai	50	16.0
Mfilou	52	16.6
Madibou	36	11.5
Djiri	35	11.2
Level of education		
Illiterate	6	1.9
Literate	307	98.1
Employment status		
Employed	268	85.6
Unemployed	45	14.4
Marital status		
Married	8	2.6
Single	298	95.2
Separated/Widow/Divorce	15	4.8
Year of diagnosis		
2022	161	51.4
2023	152	48.6
HIV/AIDS status		
Negative	298	91.7
Positive	15	5.1
History of diabetes mellitus		
No	304	97.1
Yes	9	2.9
History of TB		
New	295	94.2
Previously diagnosed	18	5.8

### Distribution of patient delay

**Fig. 1:**
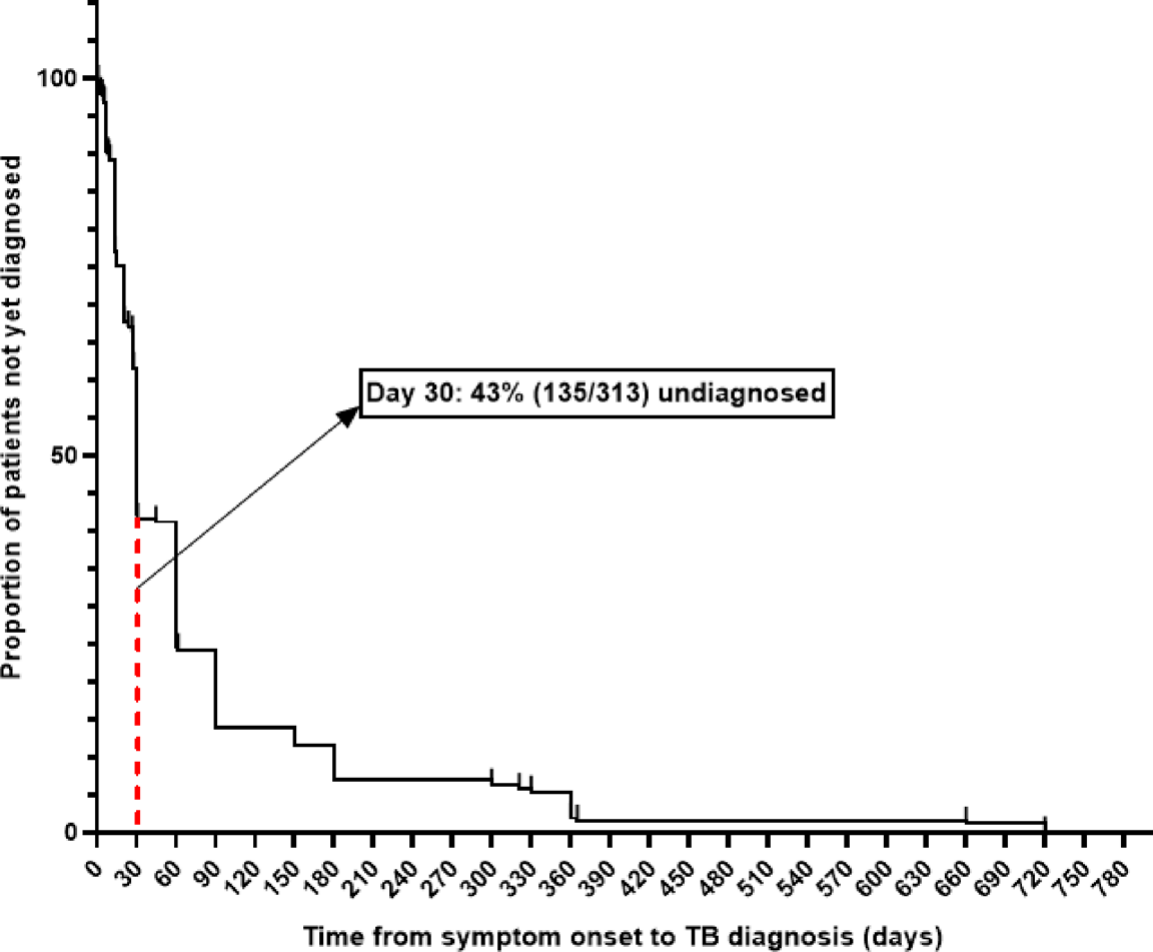
Time-to-Diagnosis curve among pulmonary tuberculosis patients. Kaplan–Meier–style curve showing the proportion of patients remaining undiagnosed over time since symptom onset. The vertical reference line at day 30 indicates that 43% (135/313) of patients had not yet been diagnosed by this time point, reflecting patient-related diagnostic delay in this setting.

The median delay from symptom onset to first presentation at the Antituberculosis Center of Brazzaville was 30 days (IQR 21–62). The longest reported delay was 720 days. Overall, 43.1% (135/313) of PTB patients experienced a delay exceeding the 30-day clinical threshold (> 30 days) (Fig. 1).

### Factors associated with patient-related diagnostic delay

In multivariate analysis, residence in Mfilou district was significantly associated with a prolonged delay (OR = 2.77, 95%CI: 1.22–6.30; *p* = 0.004). Conversely, patients with diabetes mellitus were more likely to seek care earlier (OR = 0.15, 95%CI: 0.02–1.13; *p* = 0.008). Other variables, including sex, age, marital status, employment status, education, HIV status, year of diagnosis, and TB history, showed no significant associations with diagnostic delay (Table 2).

**Table 2:** Predictors of unacceptable patient delay (> 30 days).

Variables	Delay *n* (%)	No patient delay *n* (%)	COR [95% CI]	AOR [95% CI]	*P* value
Sex					
Male	90 (41.5)	127 (58.5)	0.80 [0.49–1.30]	–	0.374
Female	45(46.9)	51 (53.1)	1		
Age group					
< 24	30 (42.3)	41 (57.7)	1		
24–44	77 (44.0)	98 (56.0)	1.07 [0.61–1.87]	–	0.802
≥ 44	28 (41.8)	39 (58.2)	0.98 [0.50–1.93]	–	0.956
Patient residence					
Makelekele	15 (31.3)	33 (68.7)	1	1	
Bacongo	13 (46.4)	15 (53.6)	1.91 [0.73–4.99]	1.88 [0.70–5.05]	0.211
Poto-Poto	5 (55.6)	4 (44.4)	2.75 [0.64–11.72]	2.61 [0.60-11.41]	0.201
Moungali	14 (48.3)	15 (51.7)	2.05 [0.79–5.31]	1.88 [0.71–4.98]	0.204
Ouenze	11 (42.3)	15 (57.7)	1.61 [0.60–4.34]	1.53 [0.56–4.21]	0.409
Talangai	22 (44.0)	28 (56.0)	1.72 [0.76–3.95]	1.90 [0.81–4.43]	0.139
Mfilou	29 (55.8)	23 (44.2)	2.77 [1.22–6.30]	3.39 [1.44–7.96]	0.004
Madibou	14 (38.9)	22 (61.1)	1.40 [0.57–3.46]	1.32 [0.52–3.35]	0.554
Djiri	12 (34.3)	23 (65.7)	1.15 [0.45–2.90]	1.14 [0.44–2.98]	0.780
Education					
Illiterate	2 (33.3)	4 (66.7)	1		
Literate	133 (43.3)	174 (56.7)	1.53 [0.28–8.47]	–	0.627
Employment status					
Employed	114 (42.5)	154 (57.5)	0.85 [0.44–1.59]	–	0.605
Unemployed	21 (46.7)	24 (53.3)	1		
Marital status					
Married	2 (25.0)	6 (75.0)	0.42 [0.08–2.12]	–	0.294
Single	130 (44.2)	164 (55.8)	1		
Separated/Widow/Divorce	3 (27.3)	8 (72.7)	0.47 [0.12–1.82]	–	0.276
Year of diagnosis					
2022	62 (38.5)	99 (61.5)	1	1	
2023	73 (48.0)	79 (52.0)	1.48 [0.94–2.31]	1.45 [0.90–2.33]	0.122
HIV/AIDS status					
Negative	133 (44.6)	165 (54.4)	1		
Positive	5 (33.3)	10 (66.7)	1.368 [0.51–3.83]	–	0.434
Diabetis mellitus					
No	134 (44.1)	170 (55.9)	1		
Yes	1 (11.1)	8 (88.9)	0.16 [0.02–1.13]	0.15 [1.01–1.27]	0.008
History of TB					
New	128 (43.4)	167 (56.6)	1		
Previously diagnosed	7 (38.9)	11 (61.1)	0.83 [0.31–2.20]	–	0.709

### Treatment outcomes and influence of patient-related diagnostic delay

Among patients with prolonged delays, 62.2% (84/135) were cured, 8.1% (11/135) completed treatment, 5.2% (7/135) died, 15.6% (21/135) were lost to follow-up, 3% (4/135) experienced treatment failure, and transfer-out accounted for 0.7% (1/135). In contrast, among those with shorter delays, 74.2% (132/178) were cured, 10.1% (18/178) completed treatment, 2.2% (4/178) died, 11.2% (20/178) were lost to follow-up, and 0.6% (1/178) experienced treatment failure. Transfer-out represented 1.1% (2/178) (Table 3).

**Table 3:** Tuberculosis treatment outcome according to the median cut-off of diagnosis delay of 30 days.

Variables	Delay *n* (%)	No patient delay *n* (%)	COR [95% CI]	AOR [95% CI]	*P* value
Treatment success					
Cured					
Yes	84 (38.9)	132 (61.1)	1.54 [0.95–2.50]	–	0.098
No	44 (49.4)	45 (50.6)	1		
Treatment completed					
Yes	11 (37.9)	18 (62.1)	1.20 [0.56–2.67]	–	0.696
No	117 (42.4)	159 (57.6)	1		
Treatment unsuccess					
Died					
Yes	7 (63.5)	4 (57.7)	1		
No	121 (41.2)	173 (58.8)	2.50 [0.78–7.76]	–	0.212
Loss to follow up					
Yes	21 (51.2)	20 (48.4)	1		
No	107 (40.5)	157 (59.5)	1.717 [0.91–3.25]	–	0.124
Treatment failure					
Yes	4 (80)	1(20)	1		
No	124 (41.3)	176 (58.7)	5.68 [0.92–69.81]	–	0.165
Transfered					
Yes	1 (33.3)	2 (66.7)	1		
No	127 (42.1)	175 (57.9)	0.69 [0.052–5.98]	–	> 0.999

Although diagnostic delay was not statistically associated with overall treatment outcome, patients with delays longer than 30 days had higher, though non-significant, odds of death (OR = 2.3, 95%CI: 0.7–7.3; *p* = 0.21) and treatment failure (OR = 5.4, 95%CI: 0.8–66.4; *p* = 0.17).

## Discussion

This study revealed that nearly half of pulmonary TB patients (43.1%) in Brazzaville experienced a diagnostic delay longer than 30 days, with a median delay of 30 days. This delay is comparable to findings from Ethiopia^12^ and Brazil^14^, where median delays of 30 days were reported. However, it is shorter than the > 45-day delays documented in Tunisia and Angola^15,16^. and considerably shorter than the 68-day delay reported in France^17^. Variations across studies may reflect differences in health system infrastructure, patient demographics, awareness of TB symptoms, and availability of diagnostic services.

Our analysis identified residence in Mfilou district as a key predictor of prolonged delay. Mfilou is located on the periphery of Brazzaville, far from the Antituberculosis Center situated in Bacongo. Distance to healthcare facilities and associated transport costs are well-recognized barriers to timely access to TB care, particularly for socioeconomically disadvantaged populations^18,19^. Additionally, limited awareness of local diagnostic services may contribute to delayed health-seeking behaviors among patients in peripheral districts.

Interestingly, patients with diabetes mellitus (DM) were more likely to be diagnosed earlier^20,21^. This finding contrasts with studies from China and other settings, where DM was associated with longer diagnostic delays. A plausible explanation is that TB patients with DM in our cohort may have presented with more severe symptoms, prompting earlier medical consultation. Alternatively, patients with chronic conditions such as DM may have more frequent contact with healthcare services, facilitating earlier detection^22^. Nevertheless, this result should be interpreted cautiously as the number of people with DM was only 9.

Contrary to expectations, HIV infection was not significantly associated with diagnostic delay. This finding is in line with some previous studies^23–26^. Our results may reflect improvements in integrated HIV/TB services and expanded access to HIV care in Congo, which could reduce barriers to TB diagnosis among people living with HIV^27–30^. This result could be the outcome of the significant progress made regarding the integrated care system for PLH^2^.

Although not statistically significant, prolonged patient-related diagnostic delay was associated with higher odds of death and treatment failure. These findings are consistent with evidence that patients diagnosed late often present with advanced disease, reduced tolerance to treatment, or poor adherence^31,32^. Late-stage disease may also limit the benefits of prolonged TB treatment regimens, contributing to poor prognosis.

Nevertheless, we observed no significant association between diagnostic delay and overall treatment outcome. This might be explained by the free access to TB diagnosis and treatment in Congo, which reduces financial barriers once patients engage with the health system. However, the higher, though non-significant, risks of death and failure among delayed patients underscore the clinical importance of addressing diagnostic delays.

Our study has some limitations. First, the definition of symptom onset relied on patient self-reporting, which is subject to recall bias. Second, we lacked data on patients’ knowledge, attitudes, and health-seeking behaviors, which may have influenced delays. Third, the relatively small number of patients with poor outcomes limited statistical power to detect significant associations. Despite these limitations, our study provides the first evidence from Congo linking diagnostic delay to treatment outcomes in TB patients. The results highlight the need for targeted interventions to improve early case detection, particularly in peripheral districts of Brazzaville. Strengthening community awareness, decentralizing diagnostic services, and addressing geographic and socioeconomic barriers could significantly reduce diagnostic delays.

## Conclusions

This study highlights a substantial diagnostic delay among pulmonary TB patients in Brazzaville, with a median time of 30 days between symptom onset and diagnostic. Residence in peripheral districts was identified as a major predictor of prolonged delay, while diabetes mellitus was unexpectedly associated with earlier diagnosis. Although diagnostic delay was not associated with treatment outcomes, patients experiencing diagnostic delays longer than 30 days showed a non-significant trend toward higher risks of death and treatment failure. These findings underscore the urgent need to strengthen case-finding strategies, improve awareness of TB symptoms, and expand access to diagnostic services in peripheral communities. Reducing diagnostic delays will be essential to improve patient outcomes and curb TB transmission in the Republic of Congo.

## Data Availability

Upon request to the corresponding author, raw data will be made available.
